# Prenatal exposure to valproic acid increases miR-132 levels in the mouse embryonic brain

**DOI:** 10.1186/s13229-017-0149-5

**Published:** 2017-06-28

**Authors:** Yuta Hara, Yukio Ago, Erika Takano, Shigeru Hasebe, Takanobu Nakazawa, Hitoshi Hashimoto, Toshio Matsuda, Kazuhiro Takuma

**Affiliations:** 1 Laboratory of Molecular Neuropharmacology, Graduate School of Pharmaceutical Sciences, Osaka University, 1-6 Yamadaoka, Suita, Osaka 565-0871 Japan; 2 Laboratory of Medicinal Pharmacology, Graduate School of Pharmaceutical Sciences, Osaka University, 1-6 Yamadaoka, Suita, Osaka 565-0871 Japan; 3 Department of Pharmacology, Graduate School of Dentistry, Osaka University, 1-8 Yamadaoka, Suita, Osaka 565-0871 Japan; 4 United Graduate School of Child Development, Osaka University, Kanazawa University, Hamamatsu University School of Medicine, Chiba University and University of Fukui, 2-2 Yamadaoka, Suita, Osaka 565-0871 Japan; 5 Division of Bioscience, Institute for Datability Science, Osaka University, 1-1 Yamadaoka, Suita, Osaka 565-0871 Japan

**Keywords:** Autism mouse model, Valproic acid, MicroRNA, Embryonic brain

## Abstract

**Background:**

MicroRNAs, small non-coding RNAs, are highly expressed in the mammalian brain, and the dysregulation of microRNA levels may be involved in neurodevelopmental disorders such as autism spectrum disorder (ASD). In the present study, we examined whether prenatal valproic acid (VPA) exposure affects levels of microRNAs, especially the brain specific and enriched microRNAs, in the mouse embryonic brain.

**Results:**

Prenatal exposure to VPA at E12.5 immediately increased miR-132 levels, but not miR-9 or miR-124 levels, in the male embryonic brain. Prenatal exposure to VPA at E12.5 also increased miR-132 levels in the female embryonic brain. We further found that the prenatal exposure to VPA at E12.5 increased mRNA levels of Arc, c-Fos and brain-derived neurotrophic factor in both male and female embryonic brains, prior to miR-132 expression. In contrast, prenatal exposure to VPA at E14.5 did not affect miR-132 levels in either male or female embryonic brain. The prenatal VPA exposure at E12.5 also decreased mRNA levels of methyl-CpG-binding protein 2 and Rho GTPase-activating protein p250GAP, both of which are molecular targets of miR-132. Furthermore, RNA sequence analysis revealed that prenatal VPA exposure caused changes in several microRNA levels other than miR-132 in the embryonic whole brain.

**Conclusions:**

These findings suggest that the alterations in neuronal activity-dependent microRNAs levels, including an increased level of miR-132, in the embryonic period, at least in part, underlie the ASD-like behaviors and cortical pathology produced by prenatal VPA exposure.

## Background

Rodents prenatally exposed to valproic acid (VPA) have been used as animal models of autism spectrum disorder (ASD) [1–3]. We recently demonstrated that mice prenatally exposed to VPA at E12.5, but not E14.5, display ASD-like behavioral abnormalities, including social interaction deficits and cognitive impairment at 8 weeks of age, and Nissl-positive cell loss in the prefrontal and somatosensory cortices after 4 weeks of age [4, 5]. Furthermore, we identified a sex difference in prenatal VPA-exposed mice, in which social interaction deficits and Nissl-positive cell loss in the somatosensory cortex are observed in only male offspring [4, 6]. However, the molecular mechanisms underlying the effects of prenatal VPA exposure remain unclear.

MicroRNAs, small non-coding RNAs, participate in post-transcriptional gene silencing via transcript degradation or translational repression in various organs and tissues [7, 8]. Recent studies have demonstrated that microRNAs play an important role in neural development in the brain [9, 10]. In addition, there is accumulating evidence that dysregulation of microRNA levels is involved in the pathogenesis of neurodevelopmental disorders, such as ASD and fragile X syndrome [11–13]. Animal studies have revealed a perturbation of microRNA levels in several brain regions in various mouse models of neurodevelopmental disorders, including methyl-CpG-binding protein 2 (MeCP2)-null mice [14, 15] and fragile X mental retardation 1 protein (*Fmr1*) knockout mice [16]. Hence, it is possible that changes in microRNA levels are involved in prenatal VPA-induced ASD-like pathology in mice. Among a variety of microRNAs, miR-132, one of brain enriched microRNAs, is shown to have important roles in the brain, axon, and synaptic development [17–21]. In addition, it is likely that miR-132 may be committed in onset of psychiatric disorders, such as depression, bipolar disorder, and schizophrenia [22–24]. Furthermore, it has also been reported that miR-9 and miR-124 are abundant in the brain and are involved in brain development [17, 25, 26].

In this study, we first investigated whether prenatal VPA exposure affects brain-enriched microRNA levels, miR-9, miR-124, and miR-132 in the male and female mouse embryonic brain. And then, we examined the effects of prenatal VPA exposure on mRNA levels of neuronal activity markers, Arc and c-Fos, brain-derived neurotrophic factor (BDNF) and miR-132 target molecules. Furthermore, we analyzed psychiatric disorder-associated microRNA levels in VPA-exposed embryonic brains by RNA sequencing.

## Methods

### Animals

Eight-week-old male and female ICR (CD1) mice were purchased from Japan SLC Inc. (Hamamatsu, Japan) and were housed in plastic cages (28 × 17 × 12 cm) under a standard light/dark cycle (12-h light cycle starting at 8:00) at a constant temperature of 22 ± 1 °C. The animals had ad libitum access to food and water, and were handled in accordance with the guidelines established by the Animal Care and Use Committee of the Graduate Schools of Pharmaceutical Sciences and Dentistry, Osaka University, the Guiding Principles for the Care and Use of Laboratory Animals approved by the Japanese Pharmacological Society, and the US National Institutes of Health Guide for the Care and Use of Laboratory Animals. The vaginal smear test was performed according to a previously published method [27, 28]. When a vaginal smear indicated proestrus or early estrus, male and female mice were mated overnight, and the next day was considered gestation day 0.

### Drug administration

The pregnant mice were intraperitoneally injected with either 500 mg/kg VPA (Sigma-Aldrich, St. Louis, MO, USA) or saline on E12.5 [4–6, 27, 28]. VPA was dissolved in 0.9% NaCl solution (Otsuka Pharma. Co., Tokushima, Japan), and the volume of injection was 10 ml/kg. All animals were returned to their home cages immediately after the injection and left undisturbed until assessment.

### Social interaction test

The social interaction test was carried out as previously described [4, 28]. Briefly, 60 min after the habituation, intruder mouse was entered into the cage. Sniffing behaviors of the test mouse to the intruder mouse was measured manually. This test was performed at 10:00–14:00.

### Sex determination

The tail of each embryo or pup was cut and transferred to a 1.5-mL microfuge tube, and 50 mM NaOH was added and heated at 95 °C for 30 min. After heating, 1 M Tris-HCl (pH 8.0) was added to each tube, and genomic DNA was subsequently extracted and used for PCR amplification. Briefly, a 0.5-μL aliquot of genomic DNA was added to the reaction mixture containing 0.625 U rTaq DNA polymerase (TOYOBO Co., Ltd., Osaka, Japan), 10× buffer (Mg^+^-free) (TOYOBO), 25 mM MgCl_2_ (TOYOBO), 2 mM dNTPs (TOYOBO), and 10 μM PCR primers. The sequences of the PCR primers are as follows: *Sry* primers, 5′-TCAAGCGCCCCATGAATGCATT-3′ (forward) and 5′-ATATTTATAGTTTGGGTATTTCTC-3′ (reverse) [29]; *Myogenin* primers, 5′-TTACGTCCATCGTGGACAGC-3′ (forward) and 5′-GCTGGGTGTTAGTCTTA-3′ (reverse) [30]. The reaction was initially heated at 94 °C for 10 min, followed by 40 cycles of 94 °C for 20 s, 53 °C for 20 s and 74 °C for 1 min. Thermocycling was performed on a TaKaRa PCR Thermal Cycler Dice (Takara Bio Inc., Otsu, Japan). The sizes of the amplified PCR products are 209 bp (*Sry*) and 246 bp (*Myogenin*). The products were separated on 2% agarose gels, and the bands were visualized with an UV transilluminator (WiseUv WUV-M20; Atto Co., Tokyo, Japan) and detected by a light-capture cooled CCD camera system (AE-6981; Atto Co.).

### Real-time PCR

Total RNA, including microRNA, was isolated from the whole brain (E12–13) of each embryo or the cerebral cortex (E18 and P1) of each pup using the miRNeasy Mini Kit (Qiagen, Hilden, Germany), and concentration was determined using the NanoDrop ND-1000 spectrophotometer (Thermo Scientific, Wilmington, DE, USA). cDNA synthesis was performed using the miScript II RT Kit (Qiagen). Real-time quantitative PCR was performed with a CFX96 Real-Time PCR Detection System (Bio-Rad, Hercules, CA, USA) using the miScript SYBR Green PCR Kit (Qiagen). All data were normalized to GAPDH and expressed as mRNA relative change, as described previously [31–33]. The following primers were used: 5′-GAAGGAGTTTCTGCAATACAGTGAG-3′ (forward) and 5′-ACATACTGAATGATCTCCTCCTCCT-3′ (reverse) for Arc; 5′-CCCATCCTTACGGACTCCC-3′ (forward) and 5′-GAGATAGCTGCTCTACTTTGCC-3′ (reverse) for c-Fos; 5′-GATGCCGCAAACATGTCTATGA-3′ (forward) and 5′-TAATACTGTCACACACGCTCAGCTC-3′ (reverse) for BDNF; 5′-TGACTTCACGGTAACTGGGA-3′ (forward) and 5′-TTTCACCTGAACACCTTCTGATG-3′ (reverse) for MeCP2; 5′-TTGAAGTGCCCCAGGTTCTT-3′ (forward) and 5′-TATATCCCATCCACAATGCCATAC-3′ (reverse) for p250GAP; 5′-GGCAAATTCAACGGCACAGT-3′ (forward) and 5′-AGATGGTGATGGGCTTCCC-3′ (reverse) for GAPDH. Determination of miR-9, miR-124 and miR-132 levels was performed using miScript Primer Assays (Qiagen).

### RNA sequence

RNA sequence analysis was carried out as previously described [34] with minor modifications. Total RNA, including microRNA, was isolated from the whole brain of each embryo of each pup using the miRNeasy Mini Kit (Qiagen) according to the manufacturer's instructions. Equal amounts of total RNAs from each embryo were combined and sequenced using the Illumina HiSeq2500/4000 system (BGI, Beijing, China). The generated fastq files were subjected to quality control. Subsequently, the reads were aligned to Rfam and Genbank. The gene expression levels were measured based on transcripts per million (TPM). We compared the gene expression levels between the VPA- and saline-treated male embryonic brain. The false discovery rate (FDR) was used to correct for multiple testing. The significance level was set at FDR < 0.05.

### Statistical analysis

All data are expressed as the mean ± standard error of the mean (S.E.M.). Statistical analyses of the experimental data were carried out using Prism 5 for Win (GraphPad Software, San Diego, CA, USA). The significance level of differences was determined using unpaired *t* test or two-way analysis of variance (ANOVA) followed by post hoc Bonferroni’s multiple comparison test. The criterion for statistical significance was *P* < 0.05.

## Results

We examined the effect of prenatally VPA exposure on social behaviors in new-born mice. At 3 weeks of age, the VPA-treated mice displayed social interaction deficits [sniff duration (sec): prenatal saline, 58.4 ± 6.4; prenatal VPA, 34.5 ± 6.6; *p* < 0.05 analyzed by unpaired *t* test; *n* = 10/group].

**Fig. 1:**
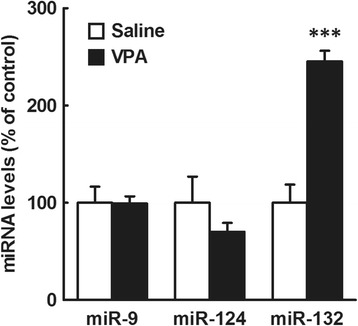
Effects of prenatal VPA exposure at E12.5 on levels of miR-9, miR-124 and miR-132 in the male mouse embryonic whole brain. Embryos were obtained from mothers treated with VPA (500 mg/kg, i.p.) or saline at E12.5 6 h after the drug exposure. Whole brains were immediately removed from the embryos, and total RNA was extracted and subjected to real-time PCR analysis. The results, which are normalized to GAPDH mRNA levels, are expressed as % of saline-treated control, and are presented as means ± SEM (*n* = 5 from two VPA- and two saline-treated dams). ^***^ *P* < 0.001, versus saline-treated control (unpaired *t* test)

We measured the levels of miR-9, miR-124 and miR-132, brain-enriched microRNAs, in the whole brain of male mouse embryos at E12.5, 6 h after VPA exposure. The VPA exposure caused an approximately 2.5-fold increase in miR-132 levels, but it did not affect the levels of miR-9 or miR-124 (n = 5/group, Fig. 1).

**Fig. 2:**
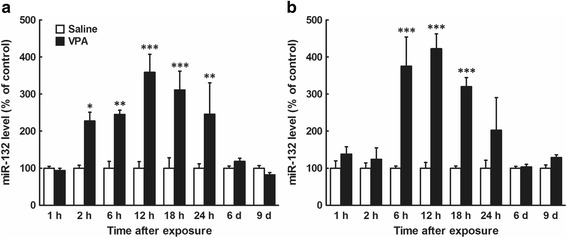
Time course of changes in miR-132 levels in male and female embryonic brains after prenatal VPA exposure at E12.5. Mothers were treated with VPA (500 mg/kg, i.p.) or saline at E12.5, and the embryos were removed at the indicated time after the drug exposure. Whole brains (E12–13) or cerebral cortex (E18 and P1) were immediately removed from the embryos or pups, and total RNA was extracted and subjected to real-time PCR analysis. The sex of each embryo was determined by PCR. **a**: Male; **b**: Female. The results, which are normalized to GAPDH mRNA levels, are expressed as % of saline-treated control, and are presented as means ± SEM (1 h, *n* = 4 from one VPA- and two saline-treated dams; 2 h, *n* = 4 from three VPA- and two saline-treated dams; 6 h, *n* = 5 from two VPA- and two saline-treated dams; 12, 18, and 24 h, *n* = 4 from two VPA- and two saline-treated dams; 6 days, *n* = 6 from three VPA- and three saline-treated dams; 9 days, *n* = 4–5 from four VPA- and two saline-treated dams). ^*^ *P* < 0.05, ^**^ *P* < 0.01, ^***^ *P* < 0.001, versus saline-treated control (two-way ANOVA followed by a post hoc Bonferroni’s multiple comparison test)

We previously found sex differences in social behavior and Nissl-positive cell numbers in the somatosensory cortex in mice prenatally exposed to VPA at E12.5 [4, 6]. Therefore, we measured changes in miR-132 levels in the brains of male and female embryos after prenatal VPA exposure at E12.5 (Fig. 2). In the male embryonic brain, two-way ANOVA revealed significant main effects of drug (*F*_1,55_ = 64.3, *P* < 0.0001) and time (*F*_7,55_ = 6.9, *P* < 0.0001), and a significant interaction between both effects (*F*_7,55_ = 6.9, *P* < 0.0001). A post hoc Bonferroni’s multiple comparison test showed a significant increase in miR-132 levels at 2 h after VPA exposure, and the increase was sustained for at least 24 h after the exposure (1, 2, 12, 18, and 24 h, *n* = 4/group; 6 h, *n* = 5/group; 6 d, *n* = 6/group; 9 d, *n* = 4–5/group; Fig. 2a). In comparison, in the female embryonic brain, prenatal VPA exposure increased miR-132 levels 6–18 h after the injection (main effects of drug [*F*_1,55_ = 54.6, *P* < 0.0001] and time [*F*_7,55_ = 7.1, *P* < 0.0001]; interaction: *F*_7,55_ = 7.1, *P* < 0.0001; 1, 2, 12, 18 and 24 h, *n* = 4/group; 6 h, *n* = 5/group; 6 d, *n* = 6/group; 9 d, *n* = 4–5/group; Fig. 2b).

**Fig. 3:**
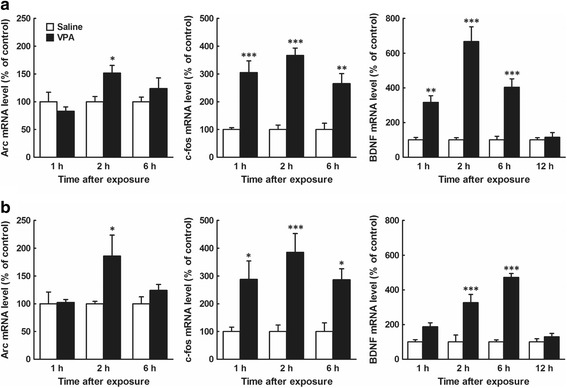
Effects of prenatal VPA exposure at E12.5 on mRNA levels of Arc, c-Fos, and BDNF in the male and female mouse embryonic whole brain. Embryos were obtained from mothers treated with VPA (500 mg/kg, i.p.) or saline at E12.5, 1, 2, 6, and 12 h after the drug exposure. Whole brains were immediately removed from the embryos, and total RNA was extracted and subjected to real-time PCR analysis. **a**: Male; **b**: Female (*left*, Arc; *middle*, c-Fos; *right*, BDNF). The results, which are normalized to GAPDH mRNA levels, are expressed as % of saline-treated control, and are presented as means ± SEM (1 h, *n* = 4 from one VPA and two saline-treated dams; 2 h, *n* = 4 from three VPA and two saline-treated dams; 6 h, *n* = 4 from two VPA and two saline-treated dams; 12 h, *n* = 4 from two VPA and two saline-treated dams). ^*^ *P* < 0.05, ^**^ *P* < 0.01, ^***^ *P* < 0.001, versus saline-treated control (two-way ANOVA followed by a post hoc Bonferroni’s multiple comparison test)

There are several studies indicating that the expression of miR-132 is induced in a neuronal activity-dependent manner [35, 36]. We next measured changes in mRNA levels of neuronal activity markers, Arc and c-Fos, and BDNF in the embryonic brains after VPA exposure. In Arc mRNA levels, two-way ANOVA revealed a significant main effect of drug (*F*_1,18_ = 5.8, *P* < 0.05) in female, but not male (*F*_1,18_ = 3.2, *P* > 0.05), and no main effect of time (male, *F*_2,18_ = 3.4, *P* > 0.05; female, *F*_2,18_ = 2.6, *P* > 0.05) and no interaction between both effects (male, *F*_2,18_ = 3.4, *P* > 0.05; female, *F*_2,18_ = 2.6, *P* > 0.05). A post hoc Bonferroni’s multiple comparison test showed a significant increase in Arc mRNA levels in the male and female embryonic brains at 2 h after the prenatal VPA exposure (*n* = 4/group; Fig. 3, *left*). In c-Fos mRNA levels, two-way ANOVA revealed a significant main effect of drug (male, *F*_1,18_ = 88.6, *P* < 0.0001; female, *F*_1,18_ = 35.0, *P* < 0.0001), but no main effect of time (male, *F*_2,18_ = 1.7, *P* > 0.05; female, *F*_2,18_ = 0.78, *P* > 0.05) and no interaction between both effects (male, *F*_2,18_ = 1.7, *P* > 0.05; female, *F*_2,18_ = 0.78, *P* > 0.05). A post hoc Bonferroni’s multiple comparison test showed a significant increase in c-Fos mRNA levels in the male and female embryonic brains at 1–6 h after the prenatal VPA exposure (*n* = 4/group; Fig. 3, *middle*). In BDNF mRNA levels, two-way ANOVA revealed significant main effects of drug (male, *F*_1,24_ = 95.6, *P* < 0.0001; female, *F*_1,24_ = 86.2, *P* < 0.0001) and time (male, *F*_3,24_ = 16.5, *P* < 0.0001; female, *F*_3,24_ = 15.8, *P* < 0.0001), and a significant interaction between both effects (male, *F*_3,24_ = 16.5, *P* < 0.0001; female, *F*_3,24_ = 15.8, *P* < 0.0001). A post hoc Bonferroni’s multiple comparison test showed a significant increase in BDNF mRNA levels in the male and female embryonic brains at 1–6 h and 2–6 h, respectively, after the prenatal VPA exposure (*n* = 4/group; Fig. 3, *right*).

**Fig. 4:**
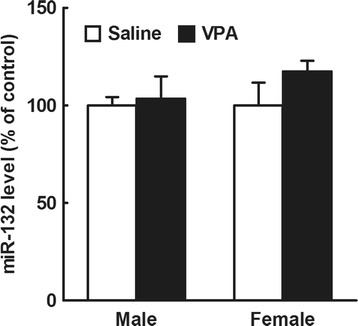
Effects of prenatal VPA exposure at E14.5 on miR-132 levels in the male and female mouse embryonic whole brain. Embryos were obtained from mothers treated with VPA (500 mg/kg, i.p.) or saline at E12.5, 6 h after the drug exposure, and whole brains were immediately removed from the embryos. Total RNA was extracted from the brains and subjected to real-time PCR analysis. The results, which are normalized to GAPDH mRNA levels, are expressed as % of saline-treated control, and are presented as means ± SEM (*n* = 4 from two VPA and one saline-treated dams)

We previously demonstrated that mice prenatally exposed to VPA at E14.5 do not display behavioral or cortical morphological abnormalities at 8 weeks of age [4]. Thus, we examined the effects of VPA exposure at E14.5 on miR-132 levels in both male and female embryonic mouse brain, and found that it had no effect (main effects of drug [*F*_1,12_ = 1.4, *P* > 0.05] and sex [*F*_1,12_ = 0.63, *P* > 0.05]; interaction: *F*_1,12_ = 0.63, *P* > 0.05 by two-way ANOVA; *n* = 4/group, Fig. 4).

**Fig. 5:**
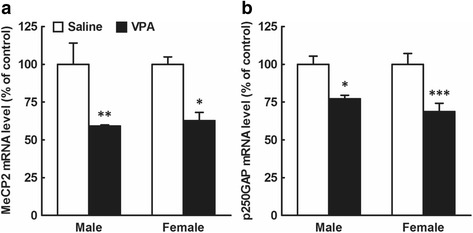
Effects of prenatal VPA exposure at E12.5 on mRNA levels of MeCP2 and p250GAP in the male and female mouse embryonic whole brain. Embryos were obtained from mothers treated with VPA (500 mg/kg, i.p.) or saline at E12.5, 6 h (**a**) or 24 h (**b**) after the drug exposure. Whole brains were immediately removed from the embryos, and total RNA was extracted and subjected to real-time PCR analysis. **a**: MeCP2. **b**: p250GAP. The results, which are normalized to GAPDH mRNA levels, are expressed as % of saline-treated control, and are presented as means ± SEM (MeCP2, *n* = 4 from two VPA and two saline-treated dams; p250GAP, *n* = 7 from two VPA- and two saline-treated dams). ^*^ *P* < 0.05, ^**^ *P* < 0.01, ^***^ *P* < 0.001, versus saline-treated control (two-way ANOVA followed by a post hoc Bonferroni’s multiple comparison test)

MeCP2 and p250GAP are targets of miR-132 [37, 38]. In this study, we examined the effects of prenatal VPA exposure at E12.5 on mRNA levels of these molecules (Fig. 5). In MeCP2 mRNA levels, two-way ANOVA revealed a significant main effect of drug (*F*_1,12_ = 24.4, *P* < 0.001), but no main effect of sex (*F*_1,12_ = 0.049, *P* > 0.05) and no interaction between both effects (*F*_1,12_ = 0.049, *P* > 0.05). A post hoc Bonferroni’s multiple comparison test showed a significant reduction in MeCP2 mRNA levels at 6 h after the prenatal VPA exposure (*n* = 4/group; Fig. 5a). In p250GAP mRNA levels, two-way ANOVA revealed a significant main effect of drug (*F*_1,24_ = 25.4, *P* < 0.0001), but no main effect of sex (*F*_1,24_ = 0.63, *P* > 0.05) and no interaction between both effects (*F*_1,24_ = 0.63, *P* > 0.05). A post hoc Bonferroni’s multiple comparison test showed a significant reduction in p250GAP mRNA levels at 24 h after the prenatal VPA exposure (n = 7/group; Fig. 5b). In contrast, there were no change in mRNA levels of MeCP2 and p250GAP in the cerebral cortex of P1 pups (MeCP2: male pups from saline-treated dams, 100.0 ± 6.5; male pups from VPA-treated dams, 105.7 ± 5.2; female pups from saline-treated dams, 100.0 ± 4.0; female pups from VPA-treated dams, 102.7 ± 4.8; main effects of drug [*F*_1,14_ = 0.62, *P* > 0.05] and sex [*F*_1,14_ = 0.076, *P* > 0.05]; interaction: *F*_1,14_ = 0.076, *P* > 0.05; analyzed with two-way ANOVA; *n* = 4–5/group; p250GAP: male pups from saline-treated dams, 100.0 ± 11.6; male pups from VPA-treated dams, 93.8 ± 5.8; female pups from saline-treated dams, 100.0 ± 3.7; female pups from VPA-treated dams, 103.2 ± 8.0; main effects of drug [*F*_1,14_ = 0.032, *P* < 0.0001] and sex [*F*_1,14_ = 0.30, *P* > 0.05]; interaction: *F*_1,14_ = 0.30, *P* > 0.05; analyzed with two-way ANOVA; *n* = 4–5/group).

We finally measured changes in microRNA levels in the embryonic brains after VPA exposure by RNA sequencing (Table 1). The prenatal VPA exposure at E12.5 caused changes in a number of psychiatric disorders-related microRNA levels including an increase in miR-132 level. It is noteworthy that RNA sequence analysis displayed no significant difference in miR-9 and miR-124 levels by the VPA exposure.

**Table 1:** Differentially expressed miRNAs, which are associated with psychiatric disorders [13], between VPA- and saline-exposed embryos in the whole brain

Name	log_2_ Ratio (VPA/Sal)	FDR
mmu-miR-15a-5p	1.76	1.86E–05
mmu-miR-15b-5p	−3.20	2.41E–41
mmu-miR-16-2-3p	−1.85	7.20E–163
mmu-miR-23b-5p	−1.30	2.75E–04
mmu-miR-24-3p	−1.29	6.11E–134
mmu-miR-30e-5p	2.55	Nearly zero
mmu-miR-34a-3p	9.68	9.53E–04
mmu-miR-92b-5p	−1.12	Nearly zero
mmu-miR-93-3p	1.34	4.00E–134
mmu-miR-96-5p	4.94	1.17E–170
mmu-miR-132-5p	1.31	3.50E–06
mmu-miR-146a-5p	1.95	2.18E–63
mmu-miR-146b-3p	−1.26	Nearly zero
mmu-miR-152-3p	1.45	Nearly zero
mmu-miR-181c-5p	−1.05	8.13E–08
mmu-miR-219a-2-3p	1.02	1.53E–276
mmu-miR-346-3p	−1.31	4.94E–12
mmu-miR-363-5p	−2.09	2.88E–29
mmu-miR-455-5p	−2.72	3.06E–238
mmu-miR-494-3p	1.04	6.20E–55

Pregnant mice were treated with VPA (500 mg/kg, i.p.) or saline on gestation days 12.5. At 12 h after the drug exposure, the whole brains were immediately removed from male embryos, total RNA was extracted, and then miRNA expression was analyzed by RNA sequencing (*n* = 3 from two VPA- and two saline-treated dams)

## Discussion

MicroRNAs have important roles in neuronal and brain development [39–42], and studies suggest that dysregulation of microRNAs contributes to neurodevelopmental disorders [11–13]. Xu et al. [43] showed that the Fragile X protein family member FXR1P regulates the levels of brain-specific miR-9 and miR-124. Abu-Elneel et al. [44] reported that miR-132 levels are downregulated in ASD postmortem brain. In addition, Lyu et al. [45] recently demonstrated that miR-132 inhibits the expression of MeCP2, mutations in which lead to Rett syndrome and autism. However, it is not known whether microRNAs play a pathogenic role in mouse models of VPA. Therefore, in the present study, we examined the effect of prenatal VPA on the levels of microRNAs in mouse embryonic brain. We found that prenatal VPA exposure at E12.5 increased miR-132 levels, but not miR-9 or miR-124 levels, in the male mouse embryonic brain. We also found that prenatal VPA exposure at E14.5 did not affect miR-132 levels. These results, taken together with the previous finding that prenatal VPA exposure at E12.5, but not E14.5, causes ASD-like behavioral abnormalities [4], suggest that dysregulation of miR-132 is involved in the pathogenesis of prenatal VPA exposure-induced ASD in the mouse.

Prenatal VPA-exposed mice show abnormal behaviors. Decreases in ultrasonic vocalization and olfactory motivation are reported at postnatal 8 to 10 days [46, 47], and social interaction deficits and cognitive impairment are observed at 8 weeks old [4, 5]. The present study shows that the decrease in social interaction is also observed at 3 weeks old. These observations suggest that alteration of psychiatric disorder-associated microRNAs including miR-132 may contribute to the behavioral phenotypes, although the exact relationship among abnormal behaviors is not known.

We previously identified sex differences in social behavior and somatosensory cortical morphology in mice exposed to VPA prenatally [4, 6]. In the present study, miR-132 levels were increased by the exposure to VPA in both male and female embryonic brains, although at 2 h, miR-132 levels were increased in males, but not yet in females. While the significance of the sex difference in the time course of the VPA-induced increase in miR-132 expression is not known, it is unlikely that dysregulation of miR-132 is related to sex differences in behavior or brain morphology.

Several studies have demonstrated that the expression of miR-132 is increased by neuronal activation [35, 36]. In this study, we found that prenatal VPA exposure increased mRNA levels of neuronal activity markers c-Fos and Arc 1–6 h and 2 h after the exposure, respectively. In addition, increase in miR-132 levels was observed in male and female embryonic brain at 2–24 and 6–18 h, respectively, after the exposure. These findings suggest that the VPA exposure-mediated neuronal activation induces an expression of miR-132. Moreover, it has also been indicated that BDNF induces miR-132 expression [37, 48, 49]. In this study, we found that prenatal VPA exposure caused an increase in BDNF mRNA levels in male and female embryonic brain at 1 and 2 h, respectively, after the exposure. It is likely that the sex difference in the time course of miR-132 levels in mouse embryos is due to BDNF mRNA levels. In contrast, the increase in BDNF mRNA levels lasted for 6 h and returned to control levels at 12 h, while the increase in miR-132 levels was sustained for at least 24 and 18 h, in male and female, respectively. Therefore, in addition to BDNF, other factors may be involved in the regulation of miR-132 levels, especially prolonged miR-132 increase. On the other hand, the plasma elimination half-life of VPA has been shown as 10–16 h [50] and 12–16 h [51]. Thus, although the exact mechanisms underlying prolonged miR-132 increase remain unclear, the length of half-life of VPA may be related to its persistency.

Several mRNAs are validated as targets of miR-132 [52]. Among them, MeCP2 and the Rho GTPase-activating protein p250GAP have been studied extensively and have been found to have key roles in neural development and dendritic spine function [53–57]. Nguyen et al. [58] suggested a critical role for MeCP2 in the maintenance of mature neuronal networks during brain development. Rietveld et al. [59] reported that layer V pyramidal neurons containing a *MeCP2* mutation have a reduced basal dendritic length and fewer branch points compared with wild-type neurons. Furthermore, it has been demonstrated that miR-132-mediated suppression of p250GAP plays a key role in dendritic plasticity [18] and hippocampal synaptogenesis [60]. In line with this observation, we found that prenatal VPA exposure at E12.5 reduced MeCP2 and p250GAP mRNA levels in the embryonic brain. Furthermore, we previously demonstrated that prenatal VPA causes a reduction in dendritic spine density in the prefrontal cortex and hippocampus [5, 28] and results in delayed maturation of primary cortical neurons prepared from mouse embryonic brains [61]. Moreover, recent study revealed that *PX-RICS* (also called as p250GAP) deletion caused various ASD-like behavioral abnormalities in mice [62]. Therefore, it is likely that the VPA-induced changes in microRNA levels, especially the increase in miR-132 levels, lead to the disruption of spinogenesis and neuronal maturation followed by the behavioral changes.

RNA sequence analysis revealed that the prenatal VPA exposure altered expression of a number of psychiatric disorder-associated microRNAs [63–65] including miR-132 in the mouse embryonic brain 12 h after the exposure. This suggests that other microRNAs other than miR-132 also play a key role in the expression of ASD-like behavioral abnormalities in the VPA-exposed mice. In this study, we did not perform behavioral analysis in the pups, but we found that the VPA-treated mice displayed social interaction deficits at 3 weeks of age. Therefore, the current study suggests that alterations of psychiatric disorder-associated microRNAs may result in the abnormal behaviors after weaning in VPA-treated mice, although it is not known they are involved in the behaviors in the pups.

## Conclusions

Prenatal VPA exposure at E12.5 increased miR-132 level, but not miR-9 and miR-124 levels, in mouse embryonic brain. The prenatal VPA exposure also caused increases in mRNA levels of c-Fos, Arc, and BDNF in both male and female embryonic brain. In addition, we demonstrated that the VPA exposure deceased mRNA levels of miR-132 target molecules. Furthermore, RNA sequence analysis revealed alterations of microRNA levels after the VPA exposure. These findings suggest that the neuronal activity-dependent changes in microRNA levels, including an increased level of miR-132, in the embryonic period are involved in the prenatal VPA-mediated ASD-like neuropathology and behavioral abnormalities.

## Abbreviations

ASD: Autism spectrum disorder
BDNF: Brain-derived neurotrophic factor
FDR: False discovery rate
MeCP2: Methyl-CpG-binding protein 2
VPA: Valproic acid
